# Adhesion G protein-coupled receptors in glioblastoma

**DOI:** 10.1093/noajnl/vdab046

**Published:** 2021-03-23

**Authors:** Gabriele Stephan, Niklas Ravn-Boess, Dimitris G Placantonakis

**Affiliations:** 1 Department of Neurosurgery, NYU Grossman School of Medicine, New York, New York, USA; 2 Kimmel Center for Stem Cell Biology, NYU Grossman School of Medicine, New York, New York, USA; 3 Laura and Isaac Perlmutter Cancer Center, NYU Grossman School of Medicine, New York, New York, USA; 4 Brain and Spine Tumor Center, NYU Grossman School of Medicine, New York, New York, USA; 5 Neuroscience Institute, NYU Grossman School of Medicine, New York, New York, USA

**Keywords:** adhesion GPCR, glioblastoma, G protein-coupled receptor

## Abstract

**Background:**

Members of the adhesion family of G protein-coupled receptors (GPCRs) have received attention for their roles in health and disease, including cancer. Over the past decade, several members of the family have been implicated in the pathogenesis of glioblastoma.

**Methods:**

Here, we discuss the basic biology of adhesion GPCRs and review in detail specific members of the receptor family with known functions in glioblastoma. Finally, we discuss the potential use of adhesion GPCRs as novel treatment targets in neuro-oncology.

## Glioblastoma

Glioma is the most common primary brain malignancy.^1^ Advances in genomics over the past decade have identified distinct driver mutations and transcriptional programs, which have led to re-classification of the glioma family. The most notable classifier in adult gliomas is neomorphic mutations in isocitrate dehydrogenase (IDH) 1 and 2. These mutations identify one type of glioma, predominantly seen in young adults, which generally has a more indolent course.^2^ IDH wild-type tumors, which include most glioblastomas (GBMs), are commonly associated with mutations in the *TERT* (telomerase) promoter and cause rapid neurologic decline and death.^3^ The Cancer Genome Atlas has used bulk transcriptome profiles to obtain gene expression signatures that further classify GBM into subtypes (classical, proneural, and mesenchymal).^4^ Similar subtypes can be derived by analyzing the DNA methylome of tumors.^5^ Nevertheless, GBM tumors display immense intratumoral heterogeneity and subtype-spanning plasticity.^6–8^ Regardless of their mutational status and transcriptome profile, gliomas are not curable by surgical excision due to their propensity to invade brain tissue.^6,9^ At the same time, gliomas, and GBM in particular, evade chemoradiotherapy through a variety of tumor cell-intrinsic and microenvironment-mediated mechanisms. Therapy resistance has been partly attributed to a cellular hierarchy dominated by stem-like cells, which are not only particularly adept at repairing DNA damage inflicted by chemoradiotherapy, but also capable of initiating tumor growth and generating all tumor lineages.^10–17^

**Figure 1.**
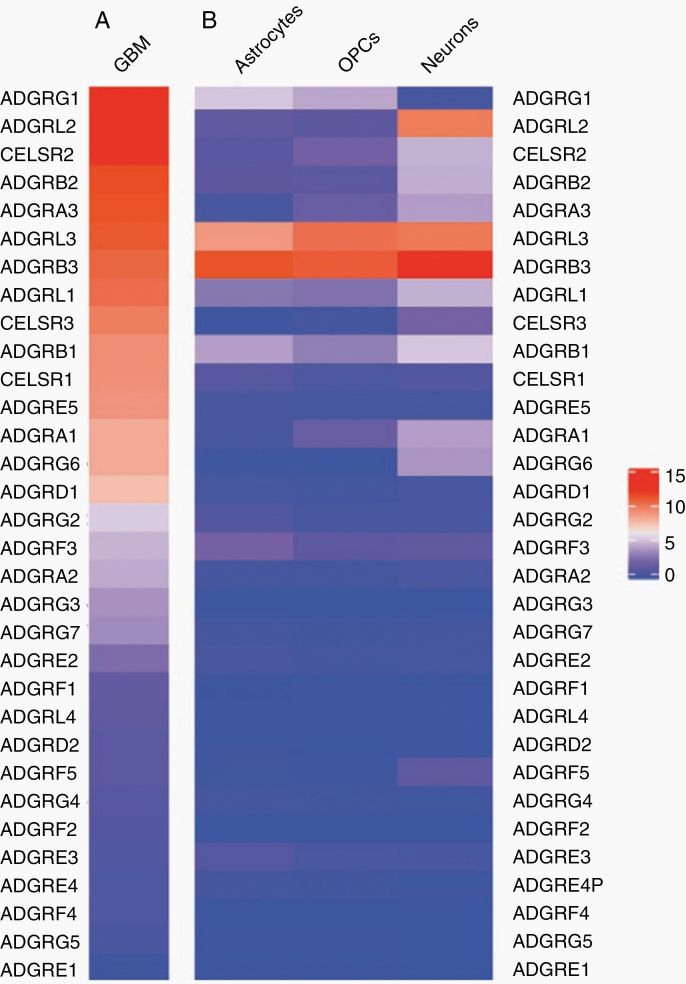
Comparison of aGPCR transcript levels in GBM and normal brain. (A) Heatmap showing ranked log_2_(FPKM) aGPCR transcript levels from averaged RNA-sequencing data of 2 patient-derived IDH wild-type GBM cultures.^18^ The 2 cultures were transcriptionally subtyped as proneural and mesenchymal and were in culture for 5 passages before sequencing. (B) Heatmap of averaged astrocyte, oligodendrocyte precursor cell (OPC), and neuron transcript level values are from Allen Brain Atlas Human Multiple Cortex Areas SMART-seq data. The ranking of aGPCRs is identical to that in (A). Data represent averaged log_2_(CPM) values from layer 1–6 cortical astrocytes (*n* = 966), layer 1–6 cortical OPCs (*n* = 773), and excitatory and inhibitory neuronal clusters (*n* = 7382). The gene expression heatmaps were generated with R.

The fact that gliomas are almost universally lethal and evade radiotherapy, conventional chemotherapy, anti-angiogenic therapy, targeted therapies, and, so far, immunotherapy, highlights the need for identifying new treatment targets. In search of such new targets, we started studying adhesion G protein-coupled receptors (aGPCRs) in GBM several years ago. As Figure 1A illustrates, analysis of our previously published RNA-sequencing data from our patient-derived GBM cultures^18^ using R indicates that several aGPCRs are expressed by tumor cells. In contrast, several of the aGPCRs expressed in GBM are absent from normal brain tissue, as evidenced by single-cell RNA-sequencing data from normal brain tissue (Allen Brain Atlas; https://celltypes.brain-map.org/rnaseq/human_m1_10x; Figure 1B). This suggests that several aGPCRs are de novo expressed in GBM. As a result, here we propose that aGPCRs may offer appealing opportunities for novel therapies in glioma.

## Classification and General Characteristics of adhesion G protein-coupled receptors

Adhesion GPCRs comprise 33 members in the human genome and represent the second largest family within the GPCR superfamily.^19,20^ According to recent classification systems, they are divided into 9 subfamilies, namely ADGRA, ADGRB, ADGRC, ADGRD, ADGRE, ADGRF, ADGRG, ADGRL, and ADGRV, although new taxonomies have recently emerged.^21^ In this review, we will primarily refer to the aGPCRs by their original names.

**Figure 2.**
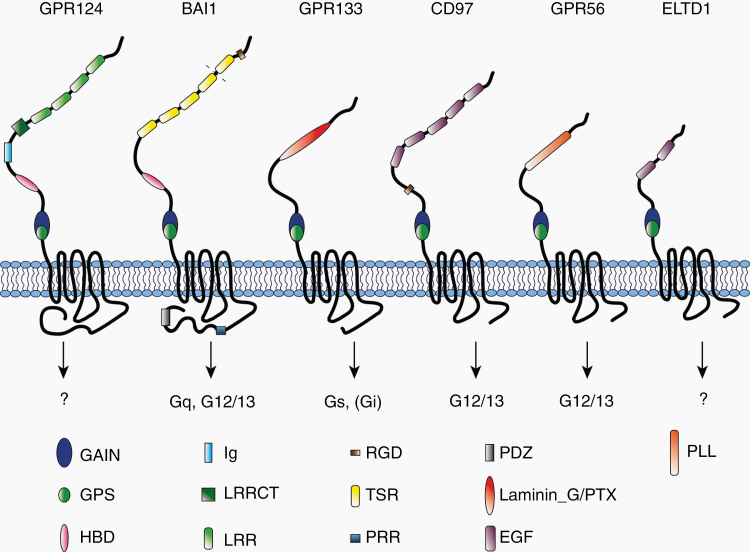
Functional domains and G protein coupling of aGPCRs implicated in GBM. The schematic shows structures and functional domains at the NTF of GPR124, BAI1, GPR133, CD97, GPR56, CELSR1, and ELTD1. G protein coupling is indicated by arrows. To date, G protein coupling of GPR124 and ELTD1 has not been documented. GAIN, GPCR autoproteolysis-inducing domain; GPS, GPCR proteolysis site; HBD, hormone-binding domain; Ig, immunoglobulin domain; LRRCT, leucine-rich repeat C-terminal domain; LRR, leucine-rich repeat; RGD, Arg-Gly-Asp motif; TSR, thrombospondin type 1 repeat; PRR, proline-rich region; laminin_G/PTX, laminin_G/pentraxin; EGF, epidermal growth factor domain; PLL, pentraxin/laminin/neurexin/sex-hormone-binding-globulin-like domain.

Like all GPCRs, members of the aGPCR family are structurally defined by 7 conserved α-helical transmembrane loops (7-TM domain), an intracellular C-terminus, and an extracellular N-terminus. What distinguishes aGPCRs from other GPCRs, however, is their long N-terminus, which varies in length and functional subdomain composition based on the receptor subtype (Figure 2). These functional domains have been shown to convey cell–cell or extracellular matrix (ECM) interactions, suggesting that these receptors have a dual role as cell adhesion and signaling proteins.^20^ All aGPCRs, with the exception of GPR123, possess a conserved GPCR autoproteolysis-inducing (GAIN) domain in the N-terminus that catalyzes cleavage at a GPCR proteolysis site (GPS) to generate an N-terminal and a C-terminal fragment (NTF and CTF, respectively).^22^ The processes following proteolysis have not been fully elucidated, but there is evidence that the NTF and CTF may remain non-covalently bound to each other in the secretory pathway and dissociate after being trafficked to the plasma membrane. Immediately distal to the GPS lies an endogenous agonist sequence, named the *Stachel* sequence, which is responsible for activating canonical signaling. Soluble peptides derived from this tethered agonist sequence have been used to experimentally modulate aGPCR function.^23–31^

To date, there are numerous publications that provide data on aGPCR canonical signaling via G proteins. Coupling to G_αs_, G_αi_, G_α12/13_, or G_αq_ proteins has been shown for many of the receptors.^20,26,29,32,33^ G protein-independent non-canonical signaling has also been reported for aGPCRs. The most prominent examples are the BAI family of aGPCRs and GPR124 (ADGRA2), which are involved in Rac-1-mediated signaling^34–36^ and Wnt pathways,^37,38^ respectively.

**Figure 3.**
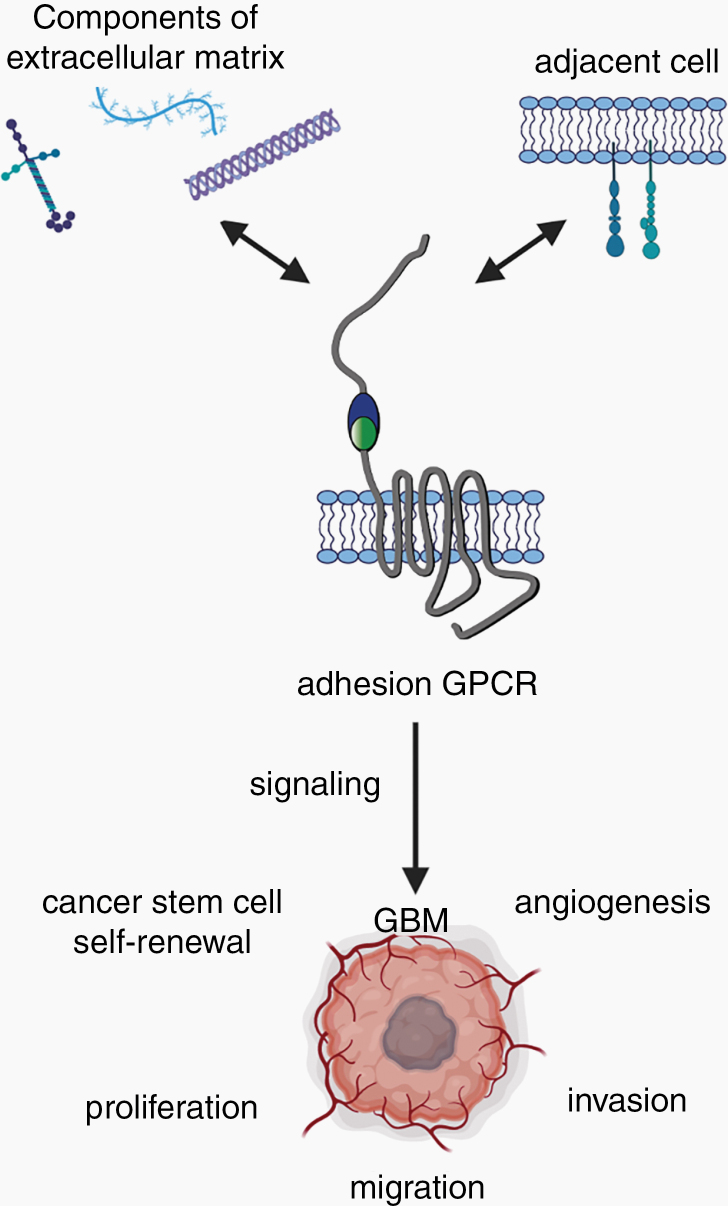
Impact of aGPCRs on GBM biology. aGPCRs bind ligands from the ECM or the plasma membrane of adjacent cells, thereby conveying cell–cell or cell–matrix interactions. Binding of a ligand results in receptor activation and either canonical signaling via G proteins or non-canonical signaling. This leads to changes in cellular processes, such as cell proliferation, migration, angiogenesis, and invasion, directly impacting GBM biology.

aGPCRs play pivotal roles in physiological cellular processes, such as establishing cell shape and polarity, mediating cell adhesion and migration, and transmitting mechanical stimuli.^39–44^ At the organismal level, aGPCRs have been implicated in the immune response, endocrine and nervous system function, and tumorigenesis.^20,21,45,46^ Moreover, aGPCRs are involved in brain development, establishment of the blood–brain barrier (BBB) and regulation of brain angiogenesis, and may contribute to the stemness of GBM stem cells (Zhou 2014, Kuhnert 2000, Kuhnert 2010, Cullen 2011, Nishimori 1997, Bayin 2016).^37,40,47,48,49^ Most importantly for the purposes of this review, several members of the aGPCR family have been implicated in glioma biology. Thus, we will focus our review on specific aGPCRs relevant for GBM and analyze their function within the context of tumor cell migration, brain invasion, cellular proliferation, stem cell self-renewal, and angiogenesis (Figure 3).

## Specific Adhesion G Protein-Coupled Receptors in GBM

Several aGPCRs have been implicated in GBM tumorigenesis. Here, we will focus on some of the specific aGPCRs that our analysis indicates are upregulated in our patient-derived GBM cultures (Figure 1A) and that are most prominent in GBM research. These include GPR124, BAI1, GPR133, CD97, EMR2, GPR56, and ELTD1. Some aGPCRs, which demonstrate similar expression patterns, for example, members of the family of cadherin EGF LAG seven-pass G-type receptors, are purposely left out of the review due to a lack of relevant literature implicating them in GBM. For the aGPCRs reviewed here, we will discuss the structural and functional properties of each receptor and provide up-to-date information regarding their implication in the oncogenic process, with a particular focus on GBM. Since several of these aGPCRs are expressed in both tumor and endothelial cells, we will review their function in both cellular contexts where appropriate.

### GPR124 (ADGRA2)

GPR124 is an orphan receptor, also known as TEM5 (tumor endothelial marker 5). According to recent taxonomy arrangements, it belongs to subfamily III of aGPCRs.^20^ GPR124’s serine/threonine-rich N-terminus is characterized by leucine-rich repeats, a leucine-rich repeat C-terminal domain, an immunoglobulin (Ig) domain, and a hormone-binding domain (HBD; Figure 2). Thrombin-induced shedding cleaves the receptor at the HBD into an NTF and CTF.^50^ The association/dissociation of NTF and CTF is protein disulfide isomerase (PDI)-dependent.^50^ Thrombin-induced cleavage exposes an RGD motif that mediates cell adhesion by binding integrins.^50,51^ A more recent study also suggests that GPR124 is involved in cell adhesion via the interaction with the Rho guanine exchange factors Elmo/Dock and intersectin through its C-terminus.^52^

Numerous publications highlighted GPR124’s involvement in Wnt signaling in the brain endothelium and the receptor’s key role in angiogenesis and development of the brain vasculature.^37,38,47,53^ Both in vitro and in vivo studies suggested that GPR124 serves as a co-activator for canonical Wnt signaling via Frizzled and Lrp receptors and Wnt7a/7b.^37,38^ Additionally, GPR124 interacts with Reck, a GPI-anchored matrix metalloprotease (MMP) inhibitor, to build a signaling complex at the level of the plasma membrane, thereby contributing to Wnt signaling in brain angiogenesis and BBB formation.^54–60^

The impact of GPR124 on adult forebrain angiogenesis and the establishment of the BBB were further investigated by producing an inducible conditional knockout in mouse endothelial cells. Endothelial GPR124 deficiency led to BBB disruption, increased tumor hemorrhage, and decreased survival in a GBM mouse model.^61^ Interestingly, the proliferation capacity of cultured GBM cells was significantly reduced by both tumor cell-specific overexpression and knockdown of the receptor.^62^ Transcript levels of *GPR124* are not detected in RNA-Seq datasets from the Allen Brain Atlas in normal brain cells (Figure 1B), while it is moderately expressed in patient-derived GBM cell lines (Figure 1A).

Collectively, most data suggest that GPR124 is mainly expressed in tumor vasculature, is upregulated in GBM, and plays a major role in Wnt signaling, an important pathway for brain angiogenesis and BBB formation. GPR124 may merit further investigation as a target in GBM, because the suppression of its pro-angiogenic function is predicted to inhibit tumor growth and progression.

### BAI1 (ADGRB1)

Subfamily VII of the aGPCR family comprises the 3 brain-specific angiogenesis inhibitor (BAI) genes: BAI1, BAI2, and BAI3.^21,63^ Like all BAIs, BAI1 harbors thrombospondin type 1 repeat domains and an HBD within its N-terminus, as well as a C-terminal PDZ domain.^63^ Of all 3 BAIs, only BAI1 contains an N-terminal RDG motif, an MMP-14 site, and a C-terminal proline-rich region (Figure 2). BAI1 is involved in both canonical G protein signaling via G_αq_ and G_α12/13_^26^ and non-canonical signaling leading to Rho pathway activation, phosphorylation of ERK, and β-arrestin binding.^64^ Recently, peptides derived from BAI1’s endogenous *Stachel* sequence were designed and used to activate the receptor in neurons, where it binds Neuroligin-1, a cell-adhesion molecule found at synapses.^65^ The *Stachel* peptide-induced activation resulted in Rac-1 activation and synapse development, highlighting the role of BAI1 in synaptogenesis.^65^

The first evidence for BAI1’s involvement in GBM was given in 1997, when Nishimori et al.^48^ found that the receptor is expressed in normal brain cells, but its transcript is significantly decreased in established GBM cell lines. Several other studies agreed with those findings, observing repeated detection of BAI1 in normal glial cells at both the transcript and protein levels, while failing to detect its presence in GBM cells.^66^ Consistent with these findings is the observation that BAI1 expression decreases with rising malignancy grades in glioma tumors.^67^ RNA-seq data from our laboratory show only moderate *BAI1* expression in patient-derived GBM cells in vitro (Figure 1A), while it is one of the top 5 detected transcripts in normal brain cells from the Allen Brain Atlas (Figure 1B). A recent study suggests that BAI1 is epigenetically downregulated in GBM by hypermethylation of its promoter region.^68^ In this study, the following evidence suggested that methyl-CpG-binding domain protein 2 (MBD2), an epigenetic regulator of gene expression, is responsible for the downregulation of BAI1 in GBM: (1) treatment of GBM cells with 5-Aza-2′-deoxycytidine, a DNA demethylating agent, restores BAI1 expression; (2) chromatin immunoprecipitation shows enrichment of MBD2 at the *BAI1* promoter region; and (3) shRNA-mediated knockdown of MDB2 leads to the re-activation of BAI1 expression in glioma cells.

BAI1 can be cleaved at its GPS, autoproteolytically, resulting in a 120 kDa NTF (Vasculostatin-120), or at its MMP-14 site, resulting in a 40 kDa NTF (Vasculostatin-40). Both cleavage products have been shown to contribute to physiological processes within the brain. Vasculostatin-120 decreases intracranial glioma growth in vivo, while both Vasculostatin-120 and Vasculostatin-40 were suggested to increase anti-angiogenic and anti-tumorigenic effects in normal brain and GBM.^69–71^ In orthotopic xenografts implanted in rats, Vasculostatin-120 reduces intracranial growth of malignant gliomas and tumor vascular density, even upon a pro-angiogenic stimulus.^71^ In endothelial cells, the anti-angiogenic effect was suggested to be dependent on the surface molecule CD36.^71^ Likewise, the anti-angiogenic and anti-tumorigenic effects of full-length BAI1 were shown in xenograft models in vivo,^72^ independent of P53 expression within the tumor.^66,73^ Taken together, these findings suggest a tumor-suppressive role for BAI1 in GBM. The identification of agents that restore expression of BAI1 could potentially serve as a therapeutic tool for the treatment of GBM.

### GPR133 (ADGRD1)

According to recent taxonomy arrangements, GPR133 belongs to subfamily V of aGPCRs.^20^ In addition to the GAIN domain and the GPS, GPR133’s N-terminal ectodomain contains a laminin G/pentraxin (LMN/PTX) domain (Figure 2). As shown in other aGPCRs, the C-terminal sequence immediately following the cleavage site within the GPS represents the endogenous tethered *Stachel* agonist, which is responsible for activating GPR133 as confirmed by mutational studies.^23^ Deleting the NTF of GPR133 leads to increased receptor activity.^23^ Initial insights into GPR133 canonical signaling and G protein binding were produced by a few recent studies. Upon GPR133 heterologous expression in Cos-7 and HEK293T cells, cAMP levels increase significantly, an effect that is eliminated with G_αs_ subunit knockdown.^74–76^ This indicates that the GPR133 receptor couples with the G_αs_ subunit upon activation. GPR133 signaling is increased by administering soluble peptides derived from the endogenous *Stachel* sequence to Cos-7 cells expressing the receptor.^23^

**Figure 4.**
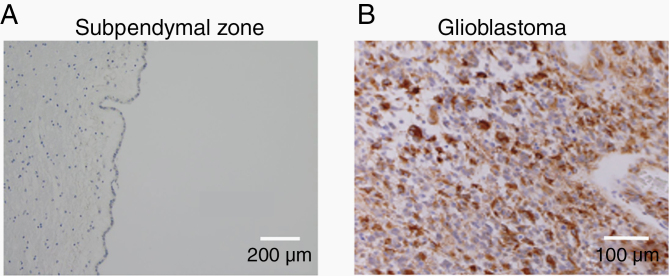
GPR133 is de novo expressed in GBM. Representative micrographs of GPR133 immunohistochemistry^78^ from the subependymal zone of non-neoplastic cadaveric brain (A) and an IDH wild-type GBM (B). The subependymal zone around the brain ventricular system contains progenitor cells that may represent the putative cell of origin for glioma.

GPR133, whose ligands remain unknown, was recently shown to be necessary for tumor growth in GBM.^49,77^ Knockdown of GPR133 by shRNA results in reduced cell proliferation and tumorsphere formation in vitro. Furthermore, GPR133 knockdown impairs orthotopic tumor xenograft initiation in vivo.^49^ RNA-Seq data from GBM cells show *GPR133* transcript expression (Figure 1A), while it is not detected in neurons, astrocytes, and oligodendrocyte precursor cells (OPCs; Figure 1B). Frenster et al.^78^ used immunohistochemistry to show that GPR133 is essentially de novo expressed in GBM, because it is absent from normal brain tissue (Figure 4). Importantly, GPR133 expression was detected in both IDH wild-type and IDH mutant tumors.^78^ Furthermore, the same study suggested a positive correlation between GPR133 expression and the WHO grade of gliomas, raising the possibility that GPR133 is a marker of anaplasia in the glioma family.

GPR133 is enriched in the most hypoxic regions of GBM, also known as areas of pseudopalisading necrosis. This phenomenon is mediated by transcriptional upregulation of *GPR133* in hypoxia via direct binding of hypoxia-inducible factor 1α (HIF-1α) to its promoter.^49^ The finding suggests that GPR133 is not only a necessary component of GBM growth, but it may also mediate the tumor’s cellular response to hypoxia. Collectively, these data suggest that GPR133 merits further consideration as a potential target in GBM. It is therefore necessary to identify inhibitory ligands, neutralizing antibodies or small molecule inhibitors of GPR133, or to engineer antibody-drug conjugates (ADCs) as therapeutics for GBM treatment.

### CD97 (ADGRE5) and EMR2 (ADGRE2)

CD97 is an aGPCR from subfamily II, consisting of 5 total EGF-TM7 receptors, aptly named for a series of epidermal growth factor (EGF) repeats found in the N-terminal ectodomain^79^ (Figure 2). In humans, the longest CD97 receptor isoform contains 5 EGF domains (EGF [1–5]), while 2 shorter isoforms result from alternative splicing and contain 3 and 4 EGF domains (EGF [1,2,5] and EGF [1,2,3,5], respectively). Autoproteolytic cleavage is observed at the GPS, which resides within the characteristic GAIN domain, and is essential for proper receptor trafficking and function.^79^

In addition to EGF domains, the NTF contains an RGD motif and several *N*-glycosylation sites. It also mediates interaction with ligands, which include CD55, chondroitin sulfate, CD90, and multiple integrins, such as α5β1.^80^ CD55 and chondroitin sulfate are both membrane-associated macromolecules expressed by immune cells and demonstrate cell-to-cell interaction with CD97.^81^ Integrin α5β1, also known as the fibronectin receptor, is a transmembrane protein that interacts with ECM components and is involved in the angiogenic process.^82^ This ligand is an attractive drug target as it is upregulated in a series of solid tumors, including GBM.^83^ The CTF of CD97 consists of the 7-TM domain and a short intracellular domain that has been shown to interact with the G_α12/13_ subunit during receptor activation. This is corroborated by RhoA activation upon ectopic CD97 expression.^84^ Nevertheless, CD97 has also been shown to couple with other G protein signaling pathways, such as G_βγ_^85^ and G_z_, where it dampens cAMP levels.^86^

CD97 has been widely studied within the context of the immune system, where the receptor facilitates migration and adhesion of leukocytes to sites of inflammation^87^ and has been implicated in the regulation of acute myeloid leukemia stem cells.^88^ New research shows an aberrant expression of the receptor in a multitude of solid tumors, including GBM.^89^ CD97 is observed to impart both an invasive and a migratory phenotype on GBM cells.^79^ CD97 expression is absent from normal brain tissue, but 2 splice variants (EGF [1,2,5] and EGF [1,2,3,5]) are highly expressed in GBM, especially in the mesenchymal and classical subtypes.^90^ Since many of the ligands that interact with CD97 do so via the EGF domains, it has been established that the different isoforms demonstrate unique ligand-binding capacities. For example, only the largest isoform recognizes chondroitin sulfate,^81^ while the smallest isoform is most likely to recognize CD55.^91^ This introduces new questions of ligand–receptor binding and how it impacts receptor processing and signaling. For example, do different ligands activate/inhibit different signaling pathways? Does the availability of a particular ligand shift isoform expression? Can interruption of a ligand–receptor interaction shift ligand preference?

EMR2 (ADGRE2) belongs to the same subfamily as CD97 and shares many similarities, including several EGF domains along its extracellular domain.^20^ High EMR2 expression has been associated with low-grade gliomas and the mesenchymal subtype of GBM.^92^ Ivan et al.^92^ found a correlation between EMR2 and the PI3K pathway, observing that both were upregulated in GBM following therapy with bevacizumab, a monoclonal antibody against VEGF-A occasionally used to treat GBM. A study has also observed that EMR2 contributes to an invasive phenotype and correlates with poor survival in GBM patients.^93^ Similar to CD97, EMR2 demonstrates binding to chondroitin sulfate.^94^

Overall, CD97 and EMR2 may represent exciting drug targets due to their high expression levels in multiple solid tumors, including GBM. Existing evidence suggests that the 2 receptors promote cellular migration and invasion, a phenotype of GBM cells linked to their aggressive behavior and poor patient prognosis. Nevertheless, growing evidence suggests that CD97 also regulates other processes, such as maintenance of the stem cell hierarchy and facilitation of cellular adhesion. Further research is needed to elucidate the function of CD97 and the impact that targeting the receptor may have on cancer progression, including in GBM.

### GPR56 (ADGRG1)

GPR56 belongs to the aGPCR subfamily VIII^20^ and is arguably the most broadly studied aGPCR within an oncological context. The receptor contains a pentraxin/laminin/neurexin/sex-hormone-binding-globulin-like (PLL) domain within its N-terminus (Figure 2), shown to be essential for ligand binding.^95^ Alternative splicing of GPR56 generates multiple isoforms, one of which is termed splice variant 4 (S4) and completely lacks the PLL domain.^95^ The receptor also contains a series of *N*- and *O*-linked glycosylation sites along its extracellular domain.^96^

Ligands of GPR56 include the ECM components collagen-III and transglutaminase-2 (TG2).^97^ Both of these proteins have been found to facilitate NTF dissociation after receptor cleavage. The binding of TG2 to the NTF of GPR56 causes the receptor–ECM complex to be internalized and degraded by the cell.^98^ GPR56, therefore, may play a role in ECM remodeling (reviewed in Ref. ^99^), which is an essential aspect of GBM cell migration and invasion. The receptor also binds heparin, a glycosaminoglycan that interacts with other ECM components.^100^ GPR56 activation has been observed in both a *Stachel*-dependent and *Stachel*-independent manner.^101^ The receptor is known to couple with the G_α12/13_ subunit to activate the Rho signaling pathway.^102^ Non-canonical signaling by GPR56 includes modulation of the PI3K/AKT^103^ and β-catenin^96^ pathways. Though GPR56 has mainly been implicated in oncogenic processes such as cellular adhesion, migration, and ECM remodeling, the receptor also seems to promote an anti-angiogenic response by reducing VEGF secretion.^104^

RNA-seq data from our laboratory show that GPR56 is the most abundantly expressed aGPCR in patient-derived GBM cells (Figure 1A), whereas single-cell SMART-seq data from the Allen Brain Atlas suggest that GPR56 expression is low in neurons and moderate in astrocytes and OPCs from normal brain tissue (Figure 1B). From the developmental point of view, GPR56 plays a crucial role in brain development, neural progenitor migration, and differentiation in the oligodendrocyte lineage and has been linked with polymicrogyria.^40,105–109^ Immunohistochemistry against GPR56 reveals its increased abundance within GBM tissue compared to normal brain tissue.^96^ The aGPCR seems to be particularly concentrated at membrane extensions (such as filopodia) and co-localizes with actin filaments at focal adhesion points within GBM cells in vitro.^96^ Moreno et al.^110^ found that GPR56 is primarily expressed in proneural and classical GBM subtypes and determined that the receptor inhibits the transition of these subtypes into the mesenchymal phenotype. While Shashidhar et al.^96^ found that GPR56 activates several oncogenic signaling cascades, including the NF-κB pathway, Moreno et al.^110^ proposed that the receptor actually inhibits the NF-κB pathway and linked its high expression in GBM tissue with better survival outcome and less radioresistance. Additional studies have found that GPR56 both suppresses and promotes cancer progression, further highlighting the conflicting role GPR56 plays in tumor biology.

Though the receptor’s exact role in GBM biology remains controversial, GPR56 serves as an important bridge facilitating connections between the extracellular and intracellular environment. Its impact on cancer progression is likely context-dependent and tissue-specific. Targeting of GPR56 with both small molecule inhibitors and monoclonal antibodies has been shown to modulate receptor signaling,^31,97^ but its overall function in GBM remains unclear.

### ELTD1 (ADGRL4)

ELTD1 (EGF, latrophilin, and 7 transmembrane domain-containing protein 1) is an aGPCR within Group I, which additionally contains 3 latrophilin receptors.^20^ Favara et al.^111^ comprehensively reviewed this receptor in 2014, detailing its structure and functions. The extracellular domain contains both an EGF domain and an EGF Ca^2+^-binding domain^112^ (Figure 2). One variant of the receptor is truncated at the C-terminal end.^111^ Currently, ELTD1 remains an orphan receptor and little is known about its posttranslational processing and signaling. In our bulk RNA-seq data, we find only modest *ELTD1* expression in patient-derived GBM cells; however, we have included it in this review due to extensive available literature implicating the receptor in GBM biology and associated angiogenesis (Figure 1A).

ELTD1 has emerged as an angiogenic biomarker, co-regulated with other angiogenic factors, such as VEGF, NOTCH1, and DLL4.^113,114^*ELTD1* is transcriptionally upregulated in blood vessels of high-grade glioma tumors compared to vessels from low-grade gliomas and from nonmalignant control tissue. Immunohistochemical analysis confirmed the expression of ELTD1 in vascular-associated cells.^113^ Li et al. demonstrated that ELTD1 acts by stimulating the JAK/STAT signaling pathway^115^ and increases the expression of HIF-1α,^116^ the transcription factor that serves as a master regulator of the hypoxic response and driver of vascularization.^117^ Since ELTD1 serves as an angiogenic marker, studies have attempted to target the receptor in the hopes of halting tumor vascularization and ultimately progression. Immunohistochemical approaches showed that ELTD1 co-localizes with the VEGF receptor (VEGFR) in mouse tumor tissue.^118^ When neutralizing antibodies were used against ELTD1, VEGFR protein levels decreased. Similarly, ELTD1 protein levels decreased upon treatment with a VEGFR neutralizing antibody.^118^ Furthermore, in vivo administration of a monoclonal antibody targeting ELTD1 reduced GBM tumor volume and microvessel density compared to an untreated control.^114^ These studies highlight the potential use of anti-ELTD1 neutralizing antibodies for anti-angiogenic therapy of GBM tumors.^114,118,119^

Studies have also demonstrated ELTD1 expression in GBM tissue itself, especially in the mesenchymal subtype, where receptor levels correlate with GBM progression and poor prognosis.^115^ Functionally, ELTD1 overexpression in established GBM cell lines promotes proliferation, migration, and invasion.^116^ Conversely, ELTD1 knockdown reduces GBM cellular viability,^120^ proliferation, and invasion capacity in vitro and decreases tumorigenesis in vivo, effects that are overcome by HIF-1α overexpression.^116^

Ultimately, neovascularization at the site of the tumor enables rapid GBM progression. Initial experiments targeting ELTD1 in mice showed promising results, effectively reducing GBM growth and vascularization. Nevertheless, clinical trials testing the effects of the anti-angiogenic drug bevacizumab on GBM outcome showed little effect on overall patient survival. It is possible that combined targeting of several angiogenic markers, such as VEGFR and ELTD1, could help reduce treatment resistance.

## Adhesion G Protein-Coupled Receptors as Potential Therapeutic Targets for Glioblastoma Treatment

Currently, no therapies targeting aGPCRs are approved or in clinical trials, although the unique features of aGPCRs show promising opportunities.^121^ In general, aGPCRs may be attractive therapeutic targets for various reasons: (1) with the exception of BAI1, aGPCRs discussed in this review are upregulated in GBM or tumor-associated vasculature compared to normal brain tissue/vasculature; (2) aGPCRs are plasma membrane proteins, which in principle makes them more accessible to BBB-permeant therapeutics relative to intracellular targets; and (3) their long extracellular N-termini with distinct domains could serve as binding sites for specific biologics. The latter feature is most relevant if the NTF and CTF of the aGPCR are associated at the plasma membrane, even after autoproteolytic cleavage of the receptor.

**Figure 5.**
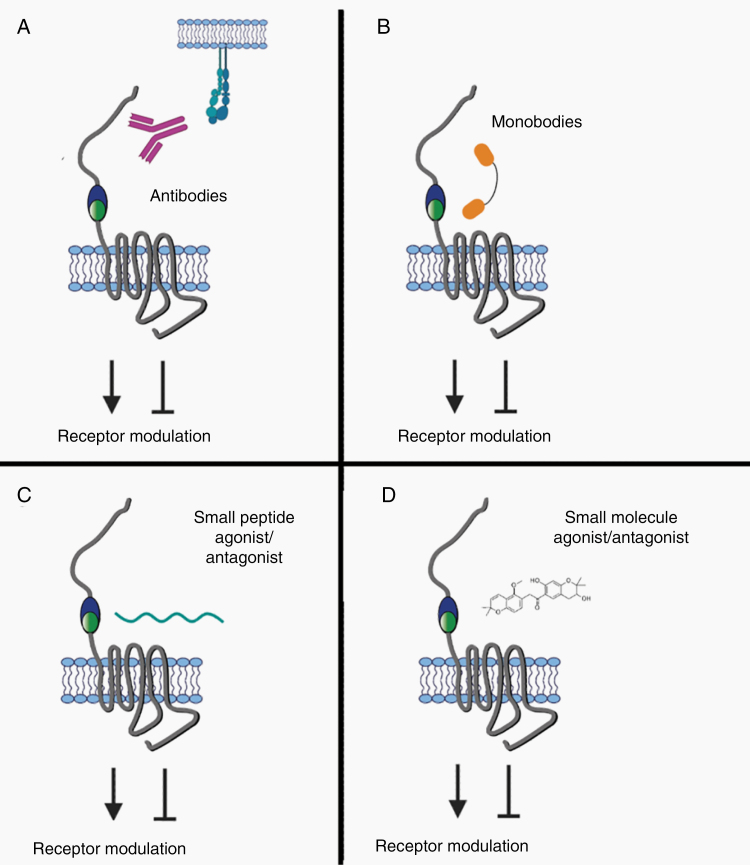
Approaches to modulating aGPCRs as targets toward novel therapeutics. (A) Antibodies that interfere with receptor–ligand interactions can modulate receptor function and signaling. Alternatively, antibodies that target the receptor and lead to internalization may be used to deliver cytotoxic cargo in ADC approaches. (B) Similar to antibodies, monobodies can modulate receptor activity or deliver cytotoxic agents upon internalization, but with the added advantage of smaller size. (C and D) Small peptide agonists and antagonists (C), derived from endogenous Stachel sequences, and small molecules (D) also represent viable approaches to modulating aGPCR signaling.

Targeting aGPCRs in GBM with antibodies could be achieved via different strategies (Figure 5). Therapeutic neutralizing antibodies may either block ligand-binding sites or otherwise prevent receptor activation and signaling. Such action could therefore inhibit aGPCR-related processes that primarily promote tumor growth and progression, such as cellular proliferation, migration, invasion, or angiogenesis. Both in vivo and in vitro experiments have demonstrated successful targeting of aGPCRs with neutralizing antibodies. In the case of ELTD1, receptor targeting with a monoclonal antibody led to reduced GBM tumor volume and vascularization in a glioma mouse model.^114^ A CD97 antibody demonstrated target specificity, cellular internalization, and safe pharmacokinetics in mice and even reduced the inflammatory response in an arthritic mouse model compared to those treated with a control,^122^ while an antibody recognizing EMR2 facilitated leukocyte migration.^39^ These examples show that antibodies targeting the extracellular domains of aGPCRs can have a range of impacts on receptor function. Another use of antibodies could be the targeted transport of cytotoxic therapeutics to tumor cells using ADCs. Since many of the aGPCRs appear to have de novo expression in GBM, they could be ideal targets for ADC approaches. No studies to date have generated ADCs targeting aGPCRs.

As an alternative to antibodies, monobodies are a novel biologic platform for targeting aGPCRs (Figure 5). Monobodies are synthetic binding proteins based on a fibronectin type III domain with an immunoglobulin fold, but without any disulfide bonds.^123^ While they can be engineered to have antibody-like target specificity, their smaller size may afford easier permeation through the BBB when GBM therapies are considered. Different monobodies against GPR56 were shown to both increase and decrease GPR56-mediated signaling in HEK239T cells and hence modulate the receptor in vitro.^101^ By targeting the PLL domain of GPR56 with monobodies, Salzman et al.^124^ were able to disrupt receptor interaction with TG2. The development of monobodies against other aGPCRs implicated in GBM and their testing in vivo would serve as an advanced tool in discovering new therapeutic options.

Targeting the ligands of aGPCRs may also be a viable approach (Figure 5). For example, a small molecule inhibitor (TTGM 5826) against TG2, a GPR56 ligand, has shown success in vitro by reducing the growth of breast cancer and GBM cells.^125^ Other promising candidates are CD55 or integrin α5β1, which serve as CD97 ligands. Knockdown of CD55 attenuated growth of prostate cancer cells^126^ and an integrin α5β1 inhibitor was successfully used to attenuate glioma growth.^127^ In fact, multiple integrin α5β1-selective biologics are currently in clinical trials (as reviewed in Refs ^128,129^). These examples help demonstrate the utility of targeting ligands toward modulating aGPCR function.

As discussed previously, peptides derived from the *Stachel* sequence have been successfully used as aGPCR agonists, modulating signaling and receptor function in vitro^21,23–31^ (Figure 5). In principle, such peptides could be mutated to inhibit aGPCR activation. However, their hydrophobic character, low solubility, and low potency currently limit possible clinical applications.

The conventional pharmacologic strategy to modulate aGPCR signaling in GBM involves small molecules, typically identified via high-throughput screening (Figure 5). To date, GPR56 and GPR114 have been successfully inhibited by the small molecule antagonist dihydromunduletone in vitro.^130^ A small molecule partial agonist of GPR56 was found to mediate G_α13_ activation.^31^ Moreover, decylubiquinone, which modulates the ROS/P53/BAI1 signaling pathway and increases BAI1 expression, reduces breast cancer growth and metastasis in a mouse model. Together, these studies suggest that the use of small molecule drugs to modulate aGPCR signaling and function is a promising approach in the treatment of cancer.^131^

In conclusion, we review compelling evidence that several aGPCRs are de novo expressed in GBM and serve primarily pro-tumorigenic roles, with the exception of BAI1, whose functional profile suggests tumor-suppressive properties. Specific aGPCRs have demonstrated direct involvement in a series of oncogenic processes, including cellular migration and invasion (CD97), stem cell self-renewal (GPR133), ECM remodeling (GPR124, GPR56, CD97), and vascularization (GPR124, BAI1, ELTD1). Given their expression profile, presence on the plasma membrane of tumor cells, potential “druggability,” and essential roles in tumorigenesis, we propose that aGPCRs represent putative novel targets in GBM. With this therapeutic potential in mind, we review exisiting data on small molecules and biologics that modulate aGPCR function and suggest opportunities for therapy development.

## Notes

**Authorship Statement.** G.S., N.R.B., and D.G.P.: Manuscript preparation.
